# Feasibility of a Novel Geriatric Rehabilitation Program for People With COPD-induced Malnutrition and Muscle Wasting: A Qualitative Study

**DOI:** 10.1177/23337214241246435

**Published:** 2024-04-28

**Authors:** Marieke Geerars-van der Veen, Judith Ballemans, Anna M. Bongers, Anouk van Loon, Ewout B. Smit

**Affiliations:** 1 Location Vrije Universiteit Amsterdam, The Netherlands; 2 Rehabilitation Center Domstate, Utrecht, The Netherlands; 3 Utrecht University, The Netherlands; 4 Amsterdam Public Health, Aging and Later Life, The Netherlands

**Keywords:** COPD, frailty, geriatric rehabilitation, malnutrition, physical activity

## Abstract

**Background and Purpose:** Patients with COPD-induced malnutrition and muscle wasting are often frail. Consequently, traditional rehabilitation may be even counterproductive due to energy costs and there is a need for specialized rehabilitation programs, which are lacking for these patients. We developed such a program, which includes resistance training, following Nonlinear Periodized Exercise principles and physical energy management, in combination with a restriction of physical activities. The purpose of the study was to investigate the feasibility and the potential effects of this program. **Methods:** Patients who are eligible for the program are those with COPD gold III/IV and a fat free mass index below standard. We conducted a qualitative feasibility study and interviewed both patients and healthcare professionals (HCPs), using a deductive approach. The open interviews were qualitatively analyzed focussing on six areas of Bowens’ feasibility model: acceptability, demand, implementation, practicality, limited efficacy, and integration. **Results and discussions:** Seven patients and seven HCPs were interviewed. For patients, key factors that helped to adhere to the program were knowledge about energy management, alternative skills to cope with COPD, and social support. They found the program beneficial. However, several patients considered a limitation of walking and ADL activities challenging. HCPs considered the program feasible and beneficial especially for those patients who accept they need a behavior change and who adhere to the program. For HCPs, key factors were the consistent approach and coaching skills of the multidisciplinary team members, and the monitoring role of the nurses. The limitation of physical activity and endurance training deviates from existing geriatric rehabilitation programs which propagate functional activity and training. Still, evidence from the current study suggests that our tailored approach for these patients might be more appropriate and also potentially effective without harm for physical function. **Conclusions:** Our novel, multidisciplinary rehabilitation program is considered feasible and clinically relevant by both patients and healthcare professionals. The next step is to explore its effects on muscle strength, physical functioning, and quality of life.

## Clinical Implications

1. Frailty is exacerbated by unintentional weight loss and exhaustion in older adults with COPD-induced malnutrition; traditional rehabilitation programs may be counterproductive due to energy costs.
2. An adapted inpatient rehabilitation program which included education and a temporarily substantially limited physical activity (except for monitored anaerobic exercise), aimed at a behavior change on energy management, rapidly improve nutrition, and promote weight gain was considered feasible by both participants and providers.
3. In frail older adults who are malnourished, weak, and fatigued, an adapted program is needed to address multiple problems concurrently.

## Introduction

Chronic Obstructive Pulmonary Disease (COPD) is the third leading cause of death worldwide (The top 10 causes of death, n.d.). It is characterized by persistent and progressive respiratory symptoms, including breathlessness or difficulty breathing, chronic cough and/or tiredness, which negatively influences the functional status and health of people with COPD (GOLD Report, 2021). Over time, patients experience worsening of the disease, mainly through more frequent and severe exacerbations.(Bousquet et al., 2015; The top 10 causes of death, n.d.) People with advanced COPD frequently face specific disabling conditions like a poor health status, comorbidities, a severely impaired functional capacity and an impaired nutritional status.(Blindenbach et al., 2017; van Dam van Isselt et al., 2014) These conditions contribute to exercise limitation, decreased endurance and physical functioning, and can result in muscle wasting. Especially COPD-induced malnutrition and muscle wasting negatively affect disease progression and prognosis, as the risk of acute exacerbations increases in these patients, leading to hospitalization (Hallin et al., 2006; Hsieh et al., 2016).

Pulmonary rehabilitation for COPD patients is considered beneficial after an exacerbation or hospitalization, if initiated within 2 weeks after discharge (Kjærgaard et al., 2020). Unfortunately, patients with advanced COPD are often not accepted into rehabilitation programs, due to their reduced exercise capacity (Blindenbach et al., 2017; van Dam van Isselt et al., 2014). For them, specialized programs have been developed, which appear to improve exercise tolerance and reduce hospital admissions (Blindenbach et al., 2017; Maddocks et al., 2016). These programs align with COPD Guidelines (GOLD Report, 2021; Vreeken et al., 2020); however, additional adaptations should be considered for patients with COPD-induced malnutrition.

Malnutrition, as defined by the Global Leadership Initiative for Malnutrition (GLIM), is associated with an increased mortality and hospitalization (Dávalos-Yerovi et al., 2021). Malnutrition in COPD patients is characterized by unintended weight loss, a low body mass index (BMI), and muscle wasting, as identified through a low fat-free mass index (FFMi) (Brighton et al., 2020; Dávalos-Yerovi et al., 2021). These patients are considered frail as they often experience one or more of the following problems: low grip strength, low energy, slowed walking speed, and low physical activity (Fried et al., 2001). By improving muscle strength, FFMi, nutritional status and energy distribution, it may be possible to interrupt the self-perpetuating circle of frailty and prevent further decline toward disability and increased frailty (Clegg, 2011). Almost two-thirds of the patients with a high symptom burden and a moderate or severe exacerbation history suffer from a low FFMi (GOLD Report, 2022; Luo et al., 2016).

Guidelines on COPD (GOLD Report, 2021; Vreeken et al., 2020) recommend endurance training but lack specific recommendations for patients with low FFMi. Frailty is exacerbated by unintentional weight loss and exhaustion in older adults with COPD-induced malnutrition and therefore traditional rehabilitation may even be counterproductive due to energy costs and extra weight loss. A specialized rehabilitation program is therefore essential because endurance training in malnourished state can even negatively affect the patients’ energy balance (Sanders et al., 2016).

Hence, we developed a novel inpatient multidisciplinary rehabilitation program specifically for these patients, based on theoretical and empirical knowledge. This program primarily includes anaerobic resistance training, following the principles on Nonlinear Periodized Exercise (NLPE) (Klijn et al., 2013), energy-management training, nutritional support, and aims to bring about behavior change. An important aspect of the program is the initial limitation of physical activities. The rational for deviating from standard rehabilitation treatment is that these patients primarily struggle with limited energy capacity, resulting in an imbalance between input and output. Temporarily limiting physical activities could help patients break the negative cycle of weight loss and recurrent exacerbations (Collins et al., 2019). It is important to note that this aspect contrasts with general geriatric and COPD guidelines, that mainly emphasize physical activity training. Furthermore, our program differs from other programs by its low intensity nature and avoidance of endurance training.

The objective of the current study was to determine the feasibility and potential benefits of the program by exploring the opinions and experiences of patients and healthcare professionals (HCPs). In particular, we were interested in evaluating the feasibility and the impact of restricting physical activities.

## Methods

### Design

To test the feasibility and potential effects of the program we conducted a qualitative study following the feasibility model of Bowen et al. (2009). Open interviews were carried out with both patients and HCPs, and analyzed with a deductive approach. The method and reporting followed the Consolidated Criteria for Reporting Qualitative Research (COREQ) (Tong et al., 2007). Since this was a feasibility study, we did not use blinding for the intervention.

### Ethical Considerations

The study received approval from the regional ethics committee for medical research, ensuring that ethical standards were met (VUmc no. 2019.726). We did not register this study in a trial database, as this was a feasibility study and not a controlled or randomized trial. The study did adhere to the standards of Good Clinical Practice, ensuring the integrity and ethical conduct of the research (ICH E6 (R2), n.d.). Participants were included in the study only after providing informed consent by signing the consent form before participating in the interviews. The whole multidisciplinary team monitored potential adverse events and reported them in the electronic patient file. Additionally, during our qualitative interview we specifically inquired about potential and actual adverse events.

### Setting

The COPD ward has 30 beds and a multidisciplinary team, including nurses on a 24-h shift. During the day, the team includes one geriatrician, three physiotherapists, one occupational therapist, one speech therapist, one dietitian, one social worker, and a one psychologist. Approximately 70% of the patients have COPD at Global Initiative for Chronic Obstructive Lung Disease (GOLD) stage III or IV (GOLD Report, 2021), with an FFMi below standard (measured by the Bodystat 1500MDD, EuroMedix), making them eligible for the program.

In The Netherlands, geriatric rehabilitation wards are primarily situated within skilled-nursing facilities. Patients can be admitted either by a geriatrician from home or directly from the hospital after receiving specialized treatment or hospitalization, provided there is an indication for low intensity (<2 hr/day) multidisciplinary treatment.

### Participants

A purposive sample of patients that followed the program was selected to participate in the study by the physiotherapists of the ward. Inclusion criteria were COPD gold III/IV and an FFMi below the standard. This was classified as an FFMi below the fifth percentile value in sex, age and BMI as compared to the reference values of the UK Biobank (Franssen et al., 2014). Exclusion criteria were: patients were cognitively incapable to understand the advice keeping up low aerobe activities, which was determined by observations of the multidisciplinary team, and scored ≤5 on the Allen Cognitive Level Screen (ACLS) (Wesson et al., 2017); patients were unable to understand the Dutch language. All patients were informed face-to-face about the study by the researcher and gave written informed consent. Also, a purposive sample of HCPs of the multidisciplinary team was selected to participate.

### Program

The program lasted between 6 to 10 weeks and consisted of three components:

#### Psychoeducation on Physical Energy Management

The team provided education on energy distribution, self-management, lifestyle, anxiety reduction, smoking cessation, and nutritional counseling, in individual and group sessions to the patients. Instructions during walking focused on important aspects like maintaining a slow walking pace, taking breaks at appropriate intervals, and probably using walking aids. Once a week the patients participated in the 1-hr group therapy “Living with a chronic disease.” In this group, themes like disease acceptance and being dependant were discussed, and exercises were conducted for emotional coping.

#### NLPE Resistance Training/Physiotherapy

Physiotherapy was offered five times a week for each patient. As part of the geriatric rehabilitation and the physiotherapy we conducted motivational interviewing and set patient-centered rehabilitation goals. Three times a week, the patients received 1-hr group session of NLPE (Klijn et al., 2013), with emphasis on anaerobic resistance training, as presented in Table 1. NLPE training forms the foundation of individualized training protocols, featuring a form of periodization where training intensity, duration and the number of sets and repetitions are altered frequently. Adaptations are provided for COPD patients with multifactorial causes of exercise intolerance. Before the NLPE training commenced, a 5-min rest period was observed during which the heart rate (HR) was measured. Following the training session another 5-min rest period was implemented, with the objective of returning the HR to its initial level. On occasions when patients experienced slow HR recovery, indicative of poor days, a longer rest period was necessary. This served as a rationale for refraining from intensifying the training regimen. Additionally, between exercises, occasional instances arose where oxygen saturation or feelings of dyspnea necessitated a slightly extended break. On two other days, a 30-min session of individual therapy took place, consisting of learning airway clearance techniques, functional transfer training and COPD education.

**Table 1.** FITT Factors of NLPE Resistance Training.

Frequency	3 Training sessions a week		
	Ratio	3 × anaerobic stimulus	1 × aerobic stimulus
Intensity	1RM range	65%	30%–40%
	Sets	3–4	2
	Repetitions	8–10	≥20
	Rest between sets	2 min	1 min
	Recovery time between exercise types	4 min	4 min
	Monitored by	Heart rate (recovery); saturation (recovery); self-ratings of Borg scale dyspnea and perceived exertion	
Type	Resistance training on leg press; seated row; chest press; leg extension		
Timing	6 weeks		

*Note.* NLPE = nonlinear periodized exercise; RM = repeated measures.

Adherence to the program was voluntary, and patients had the option to leave the ward and remain in the study. We preferred patients to use electric wheelchairs for optimal energy efficiency.

#### Limitation of Physical Activities

Upon admission the patients were encouraged to limit their activities of daily living (ADL) and functional walking, except in their room. The nurses were available 24 hr a day to help with ADL activities and mobility. An electric wheelchair was provided to cover greater distances. Every 2 weeks, the patients wore an activity monitor on the lower back (DynaPort MoveMonitor (McRoberts BV, The Hague, the Netherlands)) for three consecutive days and nights. The activity monitor provided objective information about physical activities in a patients’ daily life and the corresponding Metabolic Equivalent of Task (MET) levels (Mendes et al., 2018). Also, the patients filled in an activity diary. Both were used to provide the patients with insights to their energy expenditure, increase self-management and measure adherence to ensure that the program was followed as intended. When the body weight increased, the restrictions on physical activities were gradually lifted, which was personalized to the individual’s capabilities.

The program ended when patients were ready for the discharge, the following relative discharge criteria were: patient-centered goals were met, patient could mobilize independently, has integrated the lifestyle changes and can apply the lifestyle guidelines.

### Data Collection

Patient characteristics were collected at the week of admission. The FFMi was determined by bio-impedance analysis (Bodystat 1500MDD, EuroMedix). In addition, open in-depth interviews were conducted with both patients and HCPs, between February 2020 and June 2021. The patients were interviewed in their room, at the end of their rehabilitation period. The HCPs were interviewed during the study period. The interviews lasted between 25 and 60 min.

All interviews started with a grand tour question: “What can you tell me about your experiences with the rehabilitation program you followed?” (patients) or “What can you tell me about your experiences with the rehabilitation program on your ward?” (HCPs). This was followed by neutral open-ended questions to guide the interview. A topic list following Bowens’ feasibility criteria (Bowen et al., 2009) was used (Table 2). Directly after each interview a memo was written to capture the first impression.

**Table 2.** Key Components of Feasibility: Interview Topics and Corresponding Definitions, Based Upon Bowen et al. (2009).

Topic	Outcomes of interest	Interviews
Acceptability	How satisfied are patients and HCP with the method? Do patients intend to continue using the program? Do patients and HCP find it appropriate?	Patients and HCP
Demand	To which extend is the method needed and actually used during geriatric pulmonary rehabilitation?	Patients and HCP
	Is it likely to be used? What are positive/negative effects on the organization?	HCP
Implementation	What is necessary to implement this method?	Patients and HCP
Practicality	What are factors affecting implementation ease/difficulty, positive/negative effects on patients, perceived benefit?	Patients and HCP
Integration	What is necessary to replicate this method elsewhere, perceived fit with the infrastructure?	HCP
Limited efficacy	To what extend do the lifestyle changes made during rehabilitation transfer to the home situation, effect size estimation?	Patients and HCP

### Interview Procedure

One researcher (MG) who is an experienced interviewer and physiotherapist conducted the interviews. She was not involved in the COPD rehabilitation program. All interviews were digitally recorded (digital voice recorder Olympus WS-853) and transcribed verbatim. Insights gained during the interviews were processed and used in following interviews, leading to data-saturation. Sampling was stopped when saturation was reached: this was when all relevant feasibility topics were covered, and no new information came up from the interviews. After that, we conducted two more interviews: with one patient and one HCP, to ensure saturation.

### Data Analysis

To enhance the credibility and validity of our study, we employed several methodologic strategies. Firstly, we conducted a thematic analysis of the interviews using a deductive approach, based on Bowens’ key components of feasibility (Bowen et al., 2009). This allowed us to systematically organize and interpret the data in alignment with an established framework. Additionally, we implemented independent coding, where multiple researchers independently analyzed the data, to ensure consistency in interpretation and minimize bias. Moreover, regular research meetings were held to facilitate researcher triangulation. This enabled us to compare and discuss our individual perspectives and interpretations of the data. Furthermore, member checks were carried out with the HCP, to provide them with opportunities to review and validate the findings. These steps contributed to ensuring the robustness of our findings (Holloway & Wheeler, 2014).

Three researchers (MG, AB, (physiotherapist), ES (geriatrician, senior researcher) independently analyzed the interviews. First, all three independently wrote a memo, summarizing the most notable findings of each interview. Second, the memos were compared and discussed. The memos were used to increase the common understanding of the interviews by all three researchers. Third, couples of two researchers independently highlighted the sentences that revealed information about the key components. Fourth, all researchers compared these sentences and discussed if they revealed information about feasibility. Fifth, the selected sentences were assigned to the correct key components. The findings were summarized separately for patients and HCPs by one researcher (MG) and discussed by all three researchers. The key findings were classified according to Bowens’ feasibility model (Bowen et al., 2009) and illustrated with quotations that came from both patients and HCPs (see text and Supplemental Digital Content 1).

## Results

### Participant’s Characteristics

We conducted a total of 14 interviews, including seven patients and seven HCPs, consisting of two physiotherapists, a nurse, occupational therapist, geriatrician, dietician, and a psychologist. All eligible patients approached for the study willingly participated, with none declining. Furthermore, all participants initially enrolled remained engaged until the study’s completion. Baseline characteristics of patients and HCPs are presented in Tables 3 and 4 respectively.

**Table 3.** Patients’ Characteristics on Admission, Primary Outcomes on Admission (Pre) and After 6 Weeks (Post).

Patient	Sex (M/F)	Age (years)	Smoking status (Smoker/non-smoker)	LTOT (L/min)	GOLD (stage)	FEV1 (%predicted)		BMI (kg/m²)	Weight (kg)	FFMI^a^ (kg/m²)	LOA (days)
1	Female	72	Non-smoker	1,5	IV	23	Pre	16.7	44.1	10.8	65
							Post	17.8	47.1	12.1	
2	Male	80	Smoker	n.a.	IV	29	Pre	18.5	52.3	13.2	44
							Post	18.7	52.8	13.4	
3	Female	72	Non-smoker	n.a.	III	41	Pre	15.7	39.1	8.5	29
							Post	16.5	43.9	10.0	
4	Female	58	Smoker	n.a.	III	44	Pre	16.3	41.2	10.3	59
							Post	18.3	46.2	11.7	
5	Female	70	Non-smoker	n.a.	III	50	Pre	13.8	35.3	7.0	86
							Post	14.5	37.0	7.7	
6	Female	72	Non-smoker	n.a.	III	35	Pre	17.8	46.8	11.1	67
							Post	20.2	51.0	11.9	
7	Male	85	Non-smoker	n.a.	III	49	Pre	21.5	63.5	14.4	40
							Post	22.2	65.6	15.0	

*Note.* LTOT = long-term oxygen therapy; GOLD = global initiative for chronic obstructive lung disease; FEV1 = forced expiratory volume in 1 s; %pred = percentage of predicted; BMI = body mass index; FFMi = fat free mass index; LOA = length of admission in inpatient rehabilitation; n.a. = not applicable.

a Lowest threshold for women 15, for men 17 Sanders et al. 2016.

**Table 4.** Healthcare Professionals’ Characteristics.

Participants	Sex (M/F)	Age (years)	Profession	Years of experience with treatment of COPD patients
1	Female	27	Nurse	7
2	Female	47	Geriatrician	14
3	Female	37	Occupational therapist	9
4	Female	42	Physiotherapist	8
5	Female	35	Physiotherapist	10
6	Female	35	Psychologist	5
7	Female	56	Dietician	15

### Patient Findings

#### Acceptability and Demand

Patients considered the intensity and frequency of the physiotherapy NLPE resistance training useful and appropriate (Q1). They liked the computerized training equipment because they could train independently (Q2). One patient stated that the MoveMonitor helped her to gain insight into her actual amount of physical activity and energy expenditure (Q3). Participants had no objection to wearing the MoveMonitor. One patient complained about physical discomfort at night because of the device (Q4).

The program appeared easier to adhere to for patients who were already familiar with energy management strategies, as well as for those with such an acute or high burden of COPD that they had no alternative but to proceed cautiously (Q5 and Q6). Other patients said they found the implementation of limitation on walking and ADL activities to be more challenging than anticipated. They felt that it would have been easier for them to accept the program if they were more extensively informed about its content beforehand, than was currently the case (Q7-Q8). Most patients said it was difficult for them to be dependent (Q9). Two were afraid of physical deterioration if they would refrain from walking (Q10). One of them did not want to limit her walking and only adhered to a small part of the given advice (Q11). Another patient was not able to follow the instructions (Q12).

##### Implementation and Practicality

The patients mentioned the following benefits of the program: increased overall strength (5) (Q13), feeling more stable when walking (2) (Q14), decreased dyspnea (2), increased weight (2), decreased medication use (2) (Q15-Q17), less fear of moving (2) and improved energy management (6) (Q18 and Q19). All patients considered explanations of the method by the HCPs, social support from family and HCPs, as well as the provided tips and ADL strategies important for program adherence (Q20 and Q21).

Some patients preferred having an electric wheelchair or mobility scooter upon admission, to maintain a degree of independence, which facilitated their program adherence (Q22). Other patients were uncomfortable with dependency on “electrical propulsion” and chose not to use them (Q23).

Patients mentioned that the program required a lifestyle-shift, to adjust to better energy management (Q24 and Q25). Participating in the group therapy “Living with a chronic disease” was helpful to behavior changes, energy management and emotional coping (Q26 and Q27). However, patients mentioned little need for contact with their peers, although they sometimes informally called each other out when they saw someone overexerts himself (Q28).

##### Limited Efficacy

Patients felt confident that they could implement the required skills and benefits from the program into their home situation (Q29-Q31). One patient who had not improved on energy management skills felt reluctant to be discharged (Q32).

Key Patients’ citations:

*Q24. . . you need to accept the switchover: “I need to sit down to gain energy.” That’s how it works. Which is very strange.*
*Q31. . . Because now I know what I can and can’t do. I never used to pay attention to that, I just did everything.*

### Healthcare Professional Findings

#### Acceptability and Demand

The HCPs emphasized the necessity of the program, citing their experience that most admitted COPD patients have a low FFMi and tend to overexert themselves (Q33). The HCP recognized that the behavioral change was nearly enforced from the moment of admission, given the program’s requirement to forego functional walking and accept help with ADL activities. Initially, this appeared to provoke resistance in many patients (Q34), and it took some time for the patients to fully accept the program (Q35-Q39). Occasionally, the HCPs made compromises to the program, for instance by agreeing on a maximum number of walking episodes per day and a maximum walking distance, to make it more acceptable to the patients (Q40). This acceptance was considered part of a larger acceptation process in learning to live with COPD (Q41). The program was meant for people who are willing to change their behavior (Q42), and in such, three groups were described by the HCPs. First, the group that followed the program for the first time and followed it willingly, second, the patients who came back to follow the method again, on their own request, to brush up their knowledge regarding lifestyle or after another exacerbation (Q43), and third, a group of patients who initially had resistance to the program, refused and went home. Four HCPs considered the patients of this latter group more open to the program after another hospitalization or new exacerbation (Q44).

#### Implementation and Practicality

Several HCPs accentuated the need for a committed multidisciplinary team that is experienced in treating people with COPD (Q45). Regular training and team evaluations were needed to keep knowledge up to date and to maintain consistency (Q46). Educational and coaching skills by the team members were considered important to achieve a behavioral change in the patients and to involve formal caregivers (Q47-Q49). The 24-hr presence of the nursing staff was found crucial to supervise patients’ energy management, to take over tasks, and to give mental support (Q50). The nurses felt that an early ADL observation and advice by the occupational therapist, led to easier acceptation of help from nurses during ADL activities (Q51). Furthermore, the information derived from the Move Monitor and the activity diary helped the HCPs to coach the patients on their energy expenditure (Q52). An electric wheelchair or scooter offered patients some independent mobility, which helped to adhere to the program, although it was not applicable to all patients (Q53). All HCPs said that group therapies like “Living with a chronic disease” and the NLPE resistance training were valuable, as group dynamics helped bring about behavior change (Q54 and Q55).

The HCPs mentioned some impediments to adherence to the program: approximately 30% of the patients needed to smoke, and about 20% had a fear of exercise. Additionally, 80% of the patients had orthopedic comorbidities that sometimes prevented them from using the fitness equipment. Adjustments were made, such as using loose belts, or focussing on training arm or leg muscles only. Furthermore, in 20% of the patients, dyspnea played a role in the execution of the resistance training (Q56and Q57).

The HCPs experienced that the method was beneficial for most patients and mentioned the following effects: increased muscle strength and FFMi, positive behavior change, improved physical condition, and less hospital admissions (Q58-Q60). A minimum of 6 weeks inpatient rehabilitation was considered necessary to start with implementing behavior change, although some preferred a minimum of 8 weeks (Q61).

#### Limited Efficacy

The HCPs were convinced that the benefits of the rehabilitation tended to persevere after discharge, especially for those patients who could continue the behavior change in their home situation (Q62). There were some doubts whether all patients could implement the new behavior or that they would fall back into old patterns. They suggested a need for mono- or multidisciplinary aftercare following the same method important, to help maintain the behavior change after discharge (Q63-Q65).

#### Integration

To integrate the program, the HCPs recommended an effective day schedule for the patients, with training sessions and ADL activities spread over the day, provided with regular breaks. A training room with fitness equipment for group treatment and materials such as electric wheelchairs, oxygen equipment, nutritional products and MoveMonitors are required (Q66). For group effects, a certain volume of patients is needed (Q67). At least some of the team members should be experienced in COPD treatment (Q68).

Key HCPs’ citations:

*Q40. . .I try to compromise with the people who are extremely resistant to trying things. I tell them to try this for a week and then we will talk about it next week to see if they notice a difference. Nearly everyone says: “That actually helped.”(nurse)*

## Discussion

In this study, we provided insight into the perceptions of both patients and HCPs regarding the feasibility and potential effects of our novel multidisciplinary rehabilitation program for patients with COPD-induced malnutrition and muscle wasting. Several patients mentioned a diversity of potential effects like an overall increase in weight and strength, decreased dyspnea and improved energy management. Both patients and HCPs considered the program feasible, provided that patients commit to limiting their activities and are willing to change their behavior. Some minor program adjustments were recommended by both patients and HCPs. A significant departure from standard rehabilitation is the limitation and gradual built-up of physical activity, which we were particularly interested in assessing.

Upon admission, some patients found it challenging to limit walking and ADL activities. However, they highly appreciated and accepted the NLPE resistance training. Despite the initial challenge, patients considered the program feasible and potentially effective. For them, key factors that facilitated their acceptance and adherence included knowledge about energy management, alternative COPD coping skills, social support, and their perceived progress.

Some patients had difficulty adopting new knowledge and coping strategies, and they could not change their behavior. However, those who successfully changed their behavior during their admission felt confident about implementing these changes at home and considered the program beneficial. In line with our findings, other studies also concluded that education and skills training promote behavior change and strengthen self-management in patients with COPD. For example, Smalley et al. (2021) concluded that a patient’s willingness and ability to participate in a COPD self-management program and integrate behavior change at home after discharge can be challenging. It may be influenced by several factors; including, the ability to take on new knowledge and disease-severity. Additionally, we found that the degree of disease acceptance also plays a role in acceptance of both the program and the behavior change.

In general, the HCPs emphasized the need for a tailored rehabilitation program for patients with advanced COPD, malnutrition, and muscle-wasting. The program, especially the limitation of physical activities, was considered both feasible and potentially effective. It is important to note that this limitation poses challenges for different reasons. Firstly, it deviates from existing geriatric rehabilitation programs that promote functional activity and training. Next, patients in our study found this limitation difficult. However, due to the unique nature of COPD-related malnutrition, this restriction is considered necessary, because regular training could potentially be counterproductive. Furthermore, our ongoing study suggests that our intervention improves physical functioning despite the limitation in physical activities, making it both ethical and feasible. Nevertheless, HCPs stress the importance of providing more comprehensive information to patients before starting the program.

According to the HCPs, the potential success of the program hinged on the patients’ acceptance of the behavior change and the consistent approach and coaching skills of the multidisciplinary team members. Other key factors included the monitoring role of the nurses, peer support among the patients and the NLPE intervention.

A noteworthy finding in our study was that patients did not confirm the HCPs’ belief that group support helped them to fulfill the program. International guidelines recommend group therapy for COPD patients for several reasons, although there is limited evidence regarding the effect of peer support (Vreeken et al., 2020; Yang et al., 2021). However, our study did not find any negative effects of peer support.

The recognition and development of individualized rehabilitation programs for frail COPD patients have also been advocated by other researchers (Brighton et al., 2020; Dent et al., 2019). In line with our findings, van Dam van Isselt et al. (2014) demonstrated the feasibility and potential benefits of geriatric pulmonary rehabilitation programs for patients with advanced COPD who recently experienced an exacerbation. Self-management programs that include COPD-education and exercise information have shown positive outcomes in exercise capacity, disease management and self-efficacy, which aligns with our findings (Cannon et al., 2016; Smalley et al., 2021). Although the patients mention several potential benefits of our program, the actual effects of the program need to be explored. The benefits of NLPE resistance training itself for patients with advanced COPD were also established by Klijn et al. (2013), although our program mainly focused on the anaerobic stimulus of NLPE.

A strength of the study was the diverse range of perspectives from both patients and HCPs, which enhances the generalizability of our findings. We believe that data saturation was achieved by including and analyzing both sets of views. The use of Bowens’ feasibility framework (Bowen et al., 2009) ensured that key aspects of feasibility were assessed. Additionally, we employed data and researcher triangulation, along with member checks, to strengthen the study’s rigor (Morse et al., 2002).

A limitation of the study is the potential for performance bias, as we exclusively investigated this program within our organization. However, to the best of our knowledge, we are the first to offer such a program to patients, making it impossible to include other organizations for comparison. Another limitation is the absence of a validated tool for assessing the willingness for behavior change. Using such a tool could potentially improve patient selection. Finally, including seven patients and seven professionals could be considered a small sample. However, we specifically combined the view of both professionals and patients to determine the feasibility of our program from a broad perspective. Given the amalgamation of views along with data saturation, we are confident that we have sufficiently established the feasibility.

The current study shows that our program is feasible and appropriate for the use in clinical practice. Although the limitation of physical activity and endurance training is considered challenging, preliminary data suggest the approach may be successful. Our next step is to investigate its preliminary effects on FFMi, muscle strength, endurance capacity, ADL activities and quality of life. Subsequent research should include a cluster randomized-controlled trial to definitively determine the program’s effects.

## Conclusions

We developed a novel multidisciplinary geriatric program designed specifically for patients with advanced COPD and an FFMi below the standard. This group of patients encounters difficulties in participating in a regular rehabilitation program, and regular training may even be counterproductive. Our study found that our program is considered feasible and potentially effective by both patients and professionals. Next, the effects of our program need to be explored.

## Supplemental Material

sj-docx-1-ggm-10.1177_23337214241246435 – Supplemental material for Feasibility of a Novel Geriatric Rehabilitation Program for People With COPD-induced Malnutrition and Muscle Wasting: A Qualitative Study Supplemental material, sj-docx-1-ggm-10.1177_23337214241246435 for Feasibility of a Novel Geriatric Rehabilitation Program for People With COPD-induced Malnutrition and Muscle Wasting: A Qualitative Study by Marieke Geerars-van der Veen, Judith Ballemans, Anna M. Bongers, Anouk van Loon and Ewout B. Smit in Gerontology and Geriatric Medicine
