# Supplementary material for: Feasibility of a Novel Geriatric Rehabilitation Program for People With COPD-induced Malnutrition and Muscle Wasting: A Qualitative Study
Source: Gerontol Geriatr Med. 2024 Apr 28;10:23337214241246435. doi: 10.1177/23337214241246435 (PMC11057339; doi:10.1177/23337214241246435)
Supplement: sj-docx-1-ggm-10.1177_23337214241246435 – Supplemental material for Feasibility of a Novel Geriatric Rehabilitation Program for People With COPD-induced Malnutrition and Muscle Wasting: A Qualitative Study [file sj-docx-1-ggm-10.1177_23337214241246435.docx]

Supplemental Digital Content 1

Patients

Quotes related to acceptability and demand

| *Q1… I really enjoy the group training sessions. I could do them every day.*  *Q2… The use of the equipment. I love the fact that I can get a special bracelet* [to use the equipment] *and work out at my own pace.*  *Q3… According to my [*Move Monitor*], I’m not supposed to exercise above 3 and I was barely above 1. I did everything like I was supposed to do.*  *Q4… Sometimes I wake up and think: ‘I should change positions’ because otherwise the* [Move Monitor] *would start to irritate… Otherwise, I had no issues with it at all.*  *Q5… If I keep going when I’m tired and I don’t stop, in the end, I won’t be able to do very much. Thus, I have to rest, wherever I am.*  *Q6… The worse you feel, the more you listen to what* [HCP] *said.*  *Q7… What do they offer here… what are the guidelines in this kind of rehabilitation centre?*  *Q8… An information booklet… that includes things like this is what to expect from rehabilitation, this is what a physiotherapist does… just the daily activities. You get help showering, that kind of stuff.*  *Q9… I found* [restraining from ADL activities and walking] *difficult to accept...because I used to be so independent.*  *Q10… Looking back I find* [restraining from ADL activities and walking] *incredibly useful. However, in the beginning, I didn’t see the purpose. I didn’t really understand.*  *Q11… I would freeze or I would not be able to breathe, after all.*  *Q12… I have no patience. I really wish I had, and I say that every time. I rest a little and then I’m ready to go again. I want to do everything. I don’t want to rest.* |
| --- |

Quotes related to Practicality: perceived benefit of the program

| *Q13… When you get stronger, you can do more things. So, I do believe strength training enables you to do more.*  *Q14… I walk fairly well. I feel like my legs are stronger. I’m not as shaky anymore.*  *Q15… I suffered from shortness of breath when I came here. Now I have no issues at all and I don’t use an inhaler anymore.*  *Q16… My weight. At home, I couldn’t gain any weight, it was very difficult. It took me six months to gain a kilogram and when I started coming here, I gained a kilogram per week, which certainly helped.*  *Q17… I realised I had less shortness of breath, and I noticed I was already gaining weight*  *in the first week. I would never have been able to gain weight in that week at home.*  *Q18… Now I really do take breaks, you know. I definitely didn’t do that at home. When my body got tired, I’d just say: ‘Don’t be so sensitive and just keep going’.*  *Q19… I noticed that as long as I’m sitting down and doing something I feel like a normal person, without lung disease. However, as soon as I start moving, the fact that I have lung disease hits me. I have learned how to deal with it better.* |
| --- |

Quotes related to Implementation and Practicality: factors affecting implementation ease or difficulty

| *Q20… In the beginning I found it difficult. They would tell me: “You want to do too much all at once” and that wasn’t possible. Eventually, I understood. With showering, for example, they taught me to calmly breathe through my nose, which went surprisingly well.*  *Q21… Like sitting down while showering, for example. I’d never have thought of that myself.*  *Q22… Eventually I got an electric wheelchair... which allowed me to move on my own and I didn’t have to wait for a nurse to help me. I used to hate that.*  *Q23… Terrifying because it was so clumsy and huge and I barely weigh anything, only 37 kilograms. So that simply wasn’t doable for me.*  *Q25… Just listen. That was the most important thing for me to learn. Don’t be stubborn, accept it. When you accept your illness, you’re already 90% of the way there.*  *Q26… I went* [to the psychologist] *once and really enjoyed it. I thought about it and said to her “You know, I’m actually quite content right now”.*  *Q27… It does help. It gets you thinking so you can ask for help in time.*  *Q28… I see things happening the halls and I say ‘You can’t do that’. However, those people aren’t getting any better. I don’t know if I am visibly recovered but I feel like I am.* |
| --- |

Quotes related to Limited efficacy: transferability of the lifestyle changes to the home situation

| *Q29… I don’t think I’ll follow it one hundred percent, but I do want to stay the way I am now. When I notice that I’m having trouble breathing, or my stamina starts decreasing, I’ll have to take a break because then I’ll know that I’m doing too much.*  *Q30… I’ve learned that I shouldn’t shower alone at home anymore. It tires me too much. Now I get help at home and I have more tools so I’m able to enjoy life more. You learn that here. It won’t be over in a week, and the disease will never go away.*  *Q32… I’m just too hectic and I’m not sure what to do about it. At home it hits me so hard, and I know that everything will go wrong again tomorrow.* |
| --- |

Healthcare professionals

Quotes related to Acceptability and Demand

| *Q33… People who come from home are often used to doing too much, and they don’t realise it because they’ve taught their bodies to suppress the signals, so they just keep going. (physiotherapist 1)*  *Q34… The interesting thing is that the frustration, anger or resistance people feel is usually very informative and very helpful. It allows them to learn on the job. (psychologist)*  *Q35… During the first few weeks it’s often very confronting… we will tell them: “You are not allowed to walk”. When we are explaining muscle mass, we will typically say: “This is the norm, this is where you are, and if you walk a lot without getting the appropriate nutrition, your body will look for other sources of energy, which will usually be the protein in your muscles. Many people understand this and agree with the measures we want to take. We can’t force anyone, and we will tell them that. (physiotherapist 1)*  *Q36… I think it’s very difficult with COPD because in the early stages you’ll often get told ‘keep moving’ and eventually you reach a point where moving isn’t so good for you anymore. Of course, you do need to keep moving, but it needs to be dosed appropriately and fit with what your body can handle and that isn’t always the case. (geriatrician)*  *Q37… What I find interesting is that sitting still can truly evoke something in people that they are not used to at all. Sometimes it creates space to suddenly think and reflect. People will say; “I’m so relaxed” or “I feel safe”. Sitting still is mentally so much more than just sitting still. However, that space isn’t good for everyone, some people find out that they just can’t sit still. They become more anxious and this can also be helpful for treatment. (psychologist)*  *Q38… It takes time to sink in, and to accept it. It just takes time. (geriatrician)*  *Q39… Factors such as smoking, or wanting to do something quickly, or holding on to the feeling of independence, make it difficult for some people. (physiotherapist 1)*  *Q41… They will only progress if they stop fighting it and accept it. When they do, then they will notice things changing. We see that the acceptance process starts here in the rehabilitation centre. (psychologist)*  *Q42… So, a life in which you are no longer physically able to do what you want to do and what you have always done is hard to accept. The life one used to live is difficult to give up, due to underlying characteristics such as being caring, putting others first, perfectionism, setting the bar high, demanding a lot of yourself, etcetera. This is often accompanied by the wrong coping mechanisms such as not setting boundaries, avoiding feelings, not looking for help or support, smoking and so on. (psychologist)*  *Q43… There are people who were able to do everything well but were just sick for a very long time and they come to the point where they’ll say: ‘Can I please go back; I learned so much there.’ A group could have actually been doing very well but was just unlucky. There are various repeat patients in each group. (geriatrician)*  *Q44… If they really keep resisting and truly do not want to participate, then it’s too early for us to try to change their behaviour and they will have to go back home. It may be another year before they are ready to change. (physiotherapist 2)* |
| --- |

Quotes related to Implementation and Practicality

| \| *Q45… Our major advantage and strength is that we have an entire department*  *full of the same target group. This allows us to figure out what we see*  *often, what truly works well, the clinical added value so to say. (psychologist)*  *Q46… If we all tell the same story, it will typically be accepted. (dietician)*  *Q47… The entire team is constantly occupied with it because every time someone is*  *tired or out of breath we need to say: ‘Look, this is what is happening…’ So,*  *we just have to keep repeating all the explanations a hundred thousand times. (nurse)*  *Q48… Education is essential. Both for the patient and the family.*  *(geriatrician)*  *Q49… Definitely regarding energy management you’ll often see the family say:*  *‘You’re doing way too much, it’s not good for you.’ They are often quite happy that*  *we confirm what they’ve been feeling and saying the whole time. (geriatrician)*  *Q50… If you see that someone just keeps going or keeps on talking, you try to slow*  *them down. If you notice it, you can do something about it, but we can’t slow people down*  *one hundred percent. (nurse)*  *Q51… It makes it much more clear: someone needs help taking breaks,*  *with coaching, or physically, everything needs to be taken over. That makes it much*  *easier for people to accept. (nurse)*  *Q52… The* [Move Monitor] *obviously supports the rehabilitation process. Some people*  *need that, something visual. (occupational therapist)*  *Q53… Clearly nobody wants to be dependent. That’s why you give people an*  *electric wheelchair as soon as possible, so that they can go where they want to.*  *(physiotherapist 2)*  *Q54… The group dynamics can be very beneficial. For example, someone who’s*  *still very resistant as opposed to someone who’s been there and is now three steps further*  *and can tell the other patient: ‘I’ve been where you are. When I came here, I didn’t want*  *to hear it either, but believe me when I say that it’s the sensible thing to do’ and they’ll*  *sometimes accept it more easily coming from each other. (psychologist)*  *Q55… You will also see that they’ll try to help each other through it by sharing*  *experiences. (physiotherapist 2)*  *Q56… For example, when people can’t do a certain exercise due to*  *shoulder issues, then they are restricted to leg exercises. Everyone uses*  *the same equipment, so moving through the training area becomes difficult*  *or fewer muscle groups are trained while trying to build muscle mass.*  *Someone may have a compressed vertebra and they have to be careful or hold back*  *during training due to restrictive factors.*  *(physiotherapist 1)*  *Q57… We also see a lot of fear for shortness of breath. I think that’s something you*  *need to try to push through with strength training. For some people, it’s a fear of*  *exercising. (physiotherapist 2)* \| \| --- \| |
| --- | --- |

Quotes related to Practicality: perceived benefit of the program

| *Q58… A lot of people who come here have been underweight for a long time and they can’t improve their situation on their own. We can help those people by reducing long-distance endurance training which allows their weight to increase more effectively, and they really make significant steps. Even people who have been given feeding tubes in the hospital sometimes don’t gain weight, but by approaching it in this way, they improve by leaps and bounds. (physiotherapist 1)*  *Q59… When FFMI increases, we’ll sometimes gradually reduce walking restrictions, and as soon as someone moves more, they’ll also burn more energy so there could be a correlation when FFMI stagnates a bit. (physiotherapist 1)*  *Q60… Across the whole multidisciplinary trajectory we’ll see reduced hospitalisation afterwards. (physiotherapist 2)*  *Q61… I don’t think that you can achieve a real permanent behavioural change in six weeks. During the first week, you don’t even address behavioural changes yet. For that, people need to stay. (physiotherapist 2)* |
| --- |

Quotes related to Limited efficacy

| *Q62… If people adhere to the rules at home, continue to eat well, get enough protein, don’t burn too much energy by suddenly cleaning the whole house or walking long distances, in general, their condition can remain stable. (physiotherapist 1)*  *Q63… Of course, the method has an effect, but the more important thing is to see what the method can do in the long term… One person may pick things up faster than another or certain home caregivers are better, or people just do better in different surroundings. Aftercare is very important. (occupational therapist)*  *Q64… Sometimes home care isn’t always well designed for COPD-patients. (occupational therapist)*  *Q65… Creating awareness, also with other therapists, is certainly a challenge. (geriatrician)* |
| --- |

Quotes related to Integration

| *Q66… Electric wheelchairs and wheelchairs in general, walkers, training equipment, trained healthcare professionals. (physiotherapist)*  *Q67… Four is the maximum. You don’t want to have too many patients in a group in order to still coach a good group process. People also definitely need to be able to share their experiences with each other, so you don’t want the group to be too big. (occupational therapist)*  *Q68… A few members of the team certainly need more experience with lung problems. The program needs to be evaluated regularly. (physiotherapist 2)* |
| --- |
